# AoMae1 Regulates Hyphal Fusion, Lipid Droplet Accumulation, Conidiation, and Trap Formation in *Arthrobotrys oligospora*

**DOI:** 10.3390/jof9040496

**Published:** 2023-04-21

**Authors:** Yankun Liu, Meichen Zhu, Wenjie Wang, Xuemei Li, Na Bai, Meihua Xie, Jinkui Yang

**Affiliations:** 1 State Key Laboratory for Conservation and Utilization of Bio-Resources in Yunnan, Key Laboratory for Southwest Microbial Diversity of the Ministry of Education, School of Life Science, Yunnan University, Kunming 650032, China; 2 School of Resource, Environment and Chemistry, Chuxiong Normal University, Chuxiong 675000, China

**Keywords:** malate dehydrogenase, hyphal fusion, lipid droplet, trap formation, pathogenicity

## Abstract

Malate dehydrogenase (MDH) is a key enzyme in the tricarboxylic acid (TCA) cycle and is essential for energy balance, growth, and tolerance to cold and salt stresses in plants. However, the role of MDH in filamentous fungi is still largely unknown. In this study, we characterized an ortholog of MDH (AoMae1) in a representative nematode-trapping (NT) fungus *Arthrobotrys oligospora* via gene disruption, phenotypic analysis, and nontargeted metabolomics. We found that the loss of *Aomae1* led to a weakening of MDH activity and ATP content, a remarkable decrease in conidia yield, and a considerable increase in the number of traps and mycelial loops. In addition, the absence of *Aomae1* also caused an obvious reduction in the number of septa and nuclei. In particular, AoMae1 regulates hyphal fusion under low nutrient conditions but not in nutrient-rich conditions, and the volumes and sizes of the lipid droplets dynamically changed during trap formation and nematode predation. AoMae1 is also involved in the regulation of secondary metabolites such as arthrobotrisins. These results suggest that *Aomae1* has an important role in hyphal fusion, sporulation, energy production, trap formation, and pathogenicity in *A. oligospora*. Our results enhance the understanding of the crucial role that enzymes involved in the TCA cycle play in the growth, development, and pathogenicity of NT fungi.

## 1. Introduction

The mitochondrion is an organelle that is integrally involved in cellular energetics as well as carrying out various catabolic processes [1]. The tricarboxylic acid (TCA) cycle is the central mitochondrial metabolism that coordinates the metabolism of carbohydrates, amino acids, and fats into carbon dioxide and adenosine triphosphate (ATP) and TCA products that dynamically regulate various organizational and cellular-specific phenotypic processes [2]. In *Mycobacterium tuberculosis*, the weakening of the TCA cycle causes a thickening of the cell wall, which ultimately makes the strain more tolerant to drugs [3]. In *Staphylococcus epidermidis*, the TCA cycle induced by alkaline stress can trigger the production of reactive oxygen species (ROS) which inhibits colony growth [4]. The TCA cycle also plays an essential role in the regulation of CO_2_ sensing and mycelial development in *Candida albicans* [5]. In addition, Pb^2+^ enhances microbial activity by promoting the TCA cycle in fungi [6].

Malate dehydrogenase (MDH) is an extremely important oxidoreductase in the TCA cycle, catalyzing the dehydrogenation of L-malate to oxaloacetate. MDH further influences the synthesis and degradation of compounds by affecting the TCA cycle or the glyoxylate cycle. Examples include *Mucor circinelloides* WJ11, in which overexpression of the mitochondrial malic enzyme genes (*malC* and *malD*) improves lipid accumulation [7], and the glyoxylate cycle gene *icl1*, which is essential for the metabolic flexibility and pathogenicity of *Candida glabrata* [8]. The amino acid sequence similarity of the primary structure of MDH from different sources is minor, but the coenzyme binding, nucleotide binding, and catalytic active sites are particularly well conserved. As a critical enzyme in the central metabolic pathway of the organism, MDH has important research value in terms of genetic variation and individual development. In *Aspergillus oryzae*, the overexpression of pyruvate carboxylase and MDH enhance D-malate production [9]. In summary, MDH plays a vital role in the establishment and development of organisms, but its role in the growth and development of fungi remains largely unknown.

Nematophagous fungi are a large group of nematode antagonists widely present in various ecosystems and can be subdivided into four categories, namely, nematode-trapping (NT), endoparasitic, toxin-producing, and opportunistic fungi [10]. Of these, NT fungi can produce a variety of traps to capture and digest nematodes with a predation process that includes the attraction, adhesion, digestion, and absorption of nematodes. Trap formation is indispensable for NT fungi and is indicative of the transition from a saprophytic to a parasitic lifestyle [11,12]. *Arthrobotrys oligospora,* a model organism for the study of fungi–nematode interactions, captures nematodes by forming three-dimensional networks called traps [13]. In recent years, an increasing number of studies have revealed that signaling proteins and cellular processes, such as G-protein [14] and regulators of G-protein signaling [15,16], peroxisome [17,18], and autophagy [19,20], are involved in the regulation of mycelial growth and development, conidiation, and trap morphogenesis of *A. oligospora* and other NT fungi. In a previous study, malate synthase Mls, a key enzyme in the glyoxylate cycle, was identified in *A. oligospora*, and the deletion of *Aomls* resulted in a significant reduction in conidiation, failure to utilize fatty acids and sodium acetate for growth, and significant defects in trap morphogenesis [21]. However, the role of enzymes involved in the TCA cycle in NT fungi is largely unknown. 

In this study, AoMae1, a homologous protein of the MDH homolog, was characterized in *A. oligospora* using gene knockout, phenotypic comparison, and metabonomic analysis. We aimed to reveal the role of AoMae1 in *A. oligospora*, including mycelial growth and development, conidiation, lipid metabolism, trap formation, and pathogenicity.

## 2. Materials and Methods

### 2.1. Strains, Plasmids, and Culture Conditions

The wild-type (WT) strain *A. oligospora* (ATCC24927) and derived mutants were maintained on potato dextrose agar (PDA) at 28 °C. *Saccharomyces cerevisiae* strain FY834 was used for the construction of homologous recombinant knockout vectors and grown in yeast-extract potato dextrose (YPD, 10 g/L yeast extract, 20 g/L peptone, and 20 g/L dextrose). SC-Ura medium (2 g/L drop-out mix synthetic minus uracil without yeast nitrogen base, 26.7 g/L drop-out base with glucose, and 20 g/L agar) was used to select colonies of the yeast strain FY834 harboring correctly recombined plasmids [22]. *Escherichia coli* strain DH5a was used as a host of plasmids pRS426 and pSCN44 (Table S1). The regenerative medium PDAS (PDA supplement with 0.6 M sucrose) was used for the recovery and regeneration of protoplasts [23,24]. *Caenorhabditis elegans* was grown in oatmeal water medium at room temperature for two weeks for bioassay.

### 2.2. Bioinformatic Analysis of AoMae1

The orthologous protein of Mae1 (AoMae1, AOL_s00054g134) was retrieved from the *A. oligospora* genome based on the amino acid sequences of Mae1 from the model fungi *Neurospora crassa* (XP_011394557) and *Aspergillus nidulans* (XP_663772). The molecular weight and isoelectric point of AoMae1 were analyzed using the pI/MW tool (http://web.expasy.org/compute_pi/) (accessed 20 February 2023), and the conserved domain was analyzed using the InterProScan website (http://www.ebi.ac.uk/Tools/pfa/iprscan/) (accessed 20 February 2023). The orthologs of Mae1 from different fungi were retrieved and downloaded from the GenBank database, and the sequence similarity between AoMae1 and the other orthologs was analyzed using DNAman software (version 6). The protein sequences of Mae1 form diverse fungi were aligned with Clustalx, then, a neighbor-joining tree was constructed using the MEGA 6 software package with default parameter [25].

### 2.3. Deletion of the Aomae1 Gene

The complete fragment for gene replacement was constructed using the self-repair ability of the yeast strain FY834 [26]. The upstream and downstream fragments of the *Aomae1* gene were amplified using paired primers (Table S2), and the hygromycin resistance gene (*hph*) was obtained using pSCN44 as a template. The abovementioned three fragments and pRS426 plasmid (digested using *Eco*RI and *Xho*I) were cotransformed into the yeast FY834 strain by the PEG/CaCl_2_-mediated transformation method, and the recombinant strain was screened in SC-Ura medium to construct the knockout vector (pRS426-*Aomae1*-*hph*) [27]. The knockout fragment was amplified using the pRS426-*Aomae1*-*hph* as a template and transformed into *A. oligospora* protoplasts as described previously [26]. The putative transformants were selected on a PDAS medium supplemented with 200 μg/mL hygromycin, and positive transformants were screened and verified using PCR amplification and real-time quantitative PCR (RT-qPCR) analyses [28]. 

For PCR verification, the genomic DNAs of the WT and transformants were isolated, and a pair of primers, Mae1-PF and Mae1-PR (Table S1), located upstream and downstream of the disrupted fragment were designed and used for PCR amplification. In addition, the total RNAs of the WT and positive transformants were extracted with Trizol reagent (Invitrogen, Carlsbad, CA, USA) and reverse transcribed with a PrimeScript RT reagent kit (Takara, Shiga, Japan), using a pair of primers, Mae1-RF and Mae1-RR (Table S1), in RT-qPCR analysis [28]. The β-tubulin gene was used as an internal standard, and the relative transcript level of each gene was calculated using the threshold cycle (2^−ΔΔCT^) method.

### 2.4. Analysis of Mycelial Growth and Conidiation 

The mycelial growth of the WT and ∆*Aomae1* mutant strains was compared using PDA, tryptone glucose (TG), and tryptone yeast-extract glucose agar (TYGA) plates that were photographed on the 5th day [29,30]. The conidia yield was analyzed on a corn meal yeast extract (CMY) medium as previously described [20]. Mycelial morphology and hyphal septa were observed by staining with 20 mg/mL calcofluor white (CFW) (Sigma-Aldrich, St. Louis, MO, USA). Cell nuclei were stained with 20 mg/mL 4’,6-diamidino-2-phenylindole (DAPI, Sigma, USA) as previously described [31] and imaged under an inverted fluorescence microscope (Carl Zeiss, Oberkochen, Germany). Finally, the ultrastructure of the mycelia and spores was observed using scanning electron microscopy (SEM) [31].

### 2.5. Observation of Lipid Droplet (LD) and Hyphal Fusion

After incubation of the fungal strains on PDA medium for 5 days, mycelial samples were collected, and the LDs in hyphal cells were stained with 10 μg/mL Boron dipyrromethene dyes (BODIPY, Sigma-Aldrich) for 30 min [32]. To observe the hyphal fusion, the WT and ∆*Aomae1* mutant strains were incubated on nutrient-poor (water agar [WA, 20 g/L agar]) and nutrient-rich plates including PDA, minimal medium (MM, 0.01 g/L FeSO_4_·7 H_2_O, 20 g/L glucose, and 20 g/L agar), and WA-N (WA supplemented with 300 nematodes), respectively. Hyphae were stained with 20 mg/mL CFW and then observed using an inverted fluorescence microscope (Leica, Mannheim, Germany). In addition, the WT and mutant strains were incubated on a PDA medium for 5 days, and the LDs in mycelium were observed using transmission electron microscopy (TEM; JEM-1400Plus, Hitachi, Japan) [33].

### 2.6. Trap Induction, Pathogenicity, and Proteolytic Activity Assays

To induce trap formation, approximately 2 × 10^6^ conidia of the WT and ∆*Aomae1* mutant strains were incubated on WA plates at 28 °C for 3 days. Then, about 300 *C. elegans* individuals were introduced to each plate for trap induction and the number of traps and captured nematodes were counted at 12 h intervals. All assays were performed in triplicate. In addition, CFW and BODIPY staining were performed at 12-h intervals during trap formation and nematode predation to observe the dynamic changes of hyphal septa and accumulation of LDs. 

To analyze the proteolytic activity, the WT and ∆*Aomae1* mutant strains were inoculated in potato dextrose (PD) broth and incubated at 28 °C and 180 rpm for 5 days. The fermentation liquid was collected, and the protease activity was determined on casein plates [30].

### 2.7. Determination of MDH Activity and ATP Contents

Nicotinamide adenine dinucleotide (NADH)-dependent MDH can catalyze the reduction of oxaloacetate by NADH to produce malate. This leads to a decrease in light absorption at 340 nm, so the activity of the MDH can be determined by measuring the change in absorbance value. After the strains had been incubated in PD broth at 180 rpm for 5 days, the mycelium was filtered and frozen in liquid nitrogen for 30 min, then 0.05 g of the mycelium was weighed, and 1 mL of extraction solution was added for homogenization in an ice bath. The mixture was subsequently centrifuged at 8000× *g* at 4 °C for 10 min and the supernatant was collected for an enzyme activity assay. MDH activity was quantified using a NAD-MDH activity assay kit (BC1040, Solarbio, Beijing, China) according to the manufacturer’s protocol. 

Similarly, approximately 0.1 g of mycelium was weighed, to which 1 mL of ATP extract was added. The mixture was homogenized in an ice bath and then centrifuged at 8000× *g* at 4 °C for 10 min. The supernatant was then placed in another EP tube, 500 μL of chloroform was added, and the mixture was fully shaken. Then, the mixture was centrifuged at 10,000× *g* at 4 °C for 3 min and the supernatant was taken for an ATP content assay. Intracellular ATP was quantified using an ATP content assay kit (BC0300, Solarbio, Beijing, China) according to the manufacturer’s protocol.

### 2.8. Liquid Chromatography–Mass Spectrometry (LC−MS) Assay

The WT and mutant strains were inoculated in PD broth for 7 days at 28 °C and 180 rpm, then the mycelia and supernatant liquid were separated using vacuum filtration. The fermentation broth was shaken in an ultrasonic shaker for 30 min and three extracts were obtained by adding equal amounts of ethyl acetate. These extracts were mixed, evaporated, dried, and dissolved in chloroform: methanol = 1:1, followed by LC–MS analysis [34]. The Orbitrap mass analyzer has a full scan mode, and the scan range was 100–1000 *m*/*z*; the UV spectrum was set at 220–400 nm. The metabolic profiles of the WT and mutant strains were compared using Thermo Xcalibur software (Thermo Fisher Scientific, Miami, OK, USA). Untargeted metabolomic analysis was performed using Compound Discoverer 3.0 software (Thermo Fisher Scientific) [35]. Metabolites with differences between the WT and mutant were ranked using the variable importance in the projection score of the (O)PLS model. Those with |Log2 (fold change)| > 2 and *p*-value < 0.05 were considered to be differential metabolites between the WT strain and ∆*Aomae1* mutant [35].

### 2.9. Statistical Analysis

All experimental data were presented as the mean ± standard deviation (SD) of at least three replicated measurements. The differences between treatments were statistically evaluated by a one-way analysis of variance using Prism 9.0 (GraphPad, San Diego, CA, USA). Differences were considered statistically significant if the *p*-value < 0.05.

## 3. Results

### 3.1. Sequence Analysis of AoMae1

The AoMae1 protein contains 715 amino acid residues with an isoelectric point of 6.21 and a molecular mass of 78.32 kDa. The phylogenetic tree and multisequence alignment showed that Mae1 in *A. oligospora* shares a high degree of similarity (95.80%) with the ortholog of NT fungus *Arthrobotrys flagrans*, a moderate similarity (56.71–60.00%) with orthologs from other filamentous fungi, such as *Fusarium graminearum* (PCD40602.1) and *N. crassa* (XP_011394557.1), and a low similarity (26.98%) with *S. cerevisiae* (AJS31701.1) (Figure S1A). In addition, the MDH orthologs from different fungi contained two conserved structural domains, namely, malic-M and malic (Figure S1(Ab)).

### 3.2. AoMae1 Is Required for Hyphal Fusion under Nutrient-Deprived Conditions

**Figure 1:**
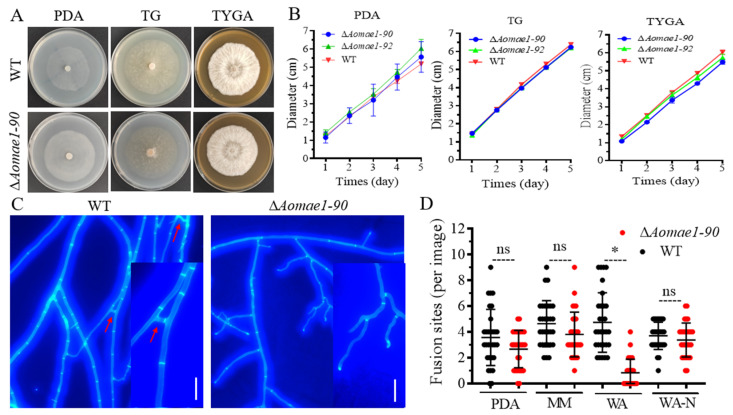
Comparison of mycelial growth and hyphal fusion between the WT and ∆*Aomae1* mutant strains. (**A**) Colony morphology of fungal strains incubated on different media for seven days at 28 °C. (**B**) Comparison of mycelial growth rate. (**C**) Observation of hyphal fusion in WA (water agar) plates. The red arrow indicates the hyphal fusion site, scale bar = 10 μm. (**D**) Comparison of the number of hyphal fusion sites under different medium conditions. The WT and mutant strains were observed using CFW staining for hyphal fusion after five days of incubation in PDA, WA, and MM media, and 30 random photographs were used to count the number of hyphal fusion sites. An asterisk indicates a significant difference between the ∆*Aomae1* mutant and the WT strain (Tukey’s HSD, * *p* < 0.05).

Two positive transformants were acquired and confirmed using PCR and RT-qPCR methods (Figure S1B,C). By comparing the colony diameter, the mycelial growth rate of the Δ*Aomae1* mutants was faster than that of the WT strain on the PDA medium, but there was no statistically significant difference between them, and the Δ*Aomae1* mutants showed a WT-like phenotype for mycelial growth on the TG medium, whereas the WT strain was slightly faster than the mutants on the TYGA medium (Figure 1A,B). In addition, CFW staining results showed that the deletion of *Aomae1* resulted in blocked mycelial fusion, which was related to nutrient availability. There was no difference in hyphal fusion between the WT and Δ*Aomae1* mutant strains on the three media of PDA, MM, and WA-N, whereas it was significantly different on the WA plate (Figure 1C,D).

### 3.3. AoMae1 Regulates Hyphal Septa, Cell Length, and the Number of Nuclei

**Figure 2:**
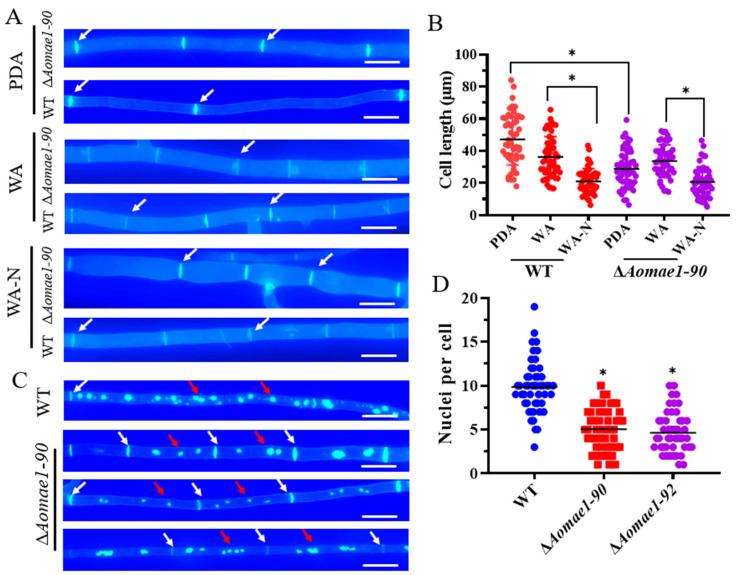
Comparison of the cell length and nuclei between WT and mutant strains. (**A**) Observation of hyphal septa of the WT strain and ∆*Aomae1* mutant cultured on PDA, WA, and WA (after nematode induction). White arrows indicate hyphal septa; scale bar = 5 μm. The mutant isolate ∆*Aomae1-90* was used as a representative strain. (**B**) Comparison of cell length of WT and ∆*Aomae1* mutant strains. The WT and mutant strains were incubated on PDA, WA, and WA-N media for five days, and 50 random photographs were used to determine the hyphal cell length. (**C**) Observation of cell nuclei of WT and ∆*Aomae1* mutant, scale bar = 5 μm. White arrows indicate hyphal septa and red arrows indicate nuclei. (**D**) Comparison of the number of nuclei. The WT and mutant strains were incubated on a PDA medium for five days, and 50 random photographs were used to determine the number of nuclei. An asterisk (**B**,**D**) indicates a significant difference between the ∆*Aomae1* mutant and the WT strain (Tukey’s HSD, * *p* < 0.05).

The deletion of *Aomae1* caused an increase in the number of hyphal septa, which resulted in a remarkable shortening of the length of mycelial cells grown on PDA medium (47.96 and 29.51 μm for the WT strain and Δ*Aomae1* mutant, respectively) (Figure 2A,B). In addition, the cell lengths varied among different media, showing PDA (47.11 ± 2.26 μm) > WA (36.19 ± 1.79) > WA-N (20.97 ± 1.12) in WT strains and WA (33.62 ± 1.43) > PDA (28.79 ± 1.68) > WA-N (20.59 ± 1.34) in the Δ*Aomae1* mutant. The average cell lengths of the WT and Δ*Aomae1* mutant strains were 36.18 and 33.62 μm on WA, respectively, whereas they were 20.97 and 20.59 μm on WA-N, respectively (Figure 2A,B). During trap formation, the cell length appeared to be shortened to a greater extent (Figure S2A,B), and the average trap cell lengths were 14.59 and 16.26 μm in the WT and ∆*Aomae1* strains, respectively. Using DAPI staining, we found that the number of nuclei varied considerably between the WT and ∆*Aomae1* strains (Figure 2C,D). In the WT strain, the number of nuclei per cell ranged from 3 to 16, with an average of 9.86 ± 0.42, whereas the ∆*Aomae1* strain had 1 to 14 nuclei, with an average of 5.14 ± 0.32 nuclei per cell (Figure 2C,D). 

### 3.4. AoMae1 Regulates the Volume of LDs during Trap Formation and Nematode Predation

**Figure 3:**
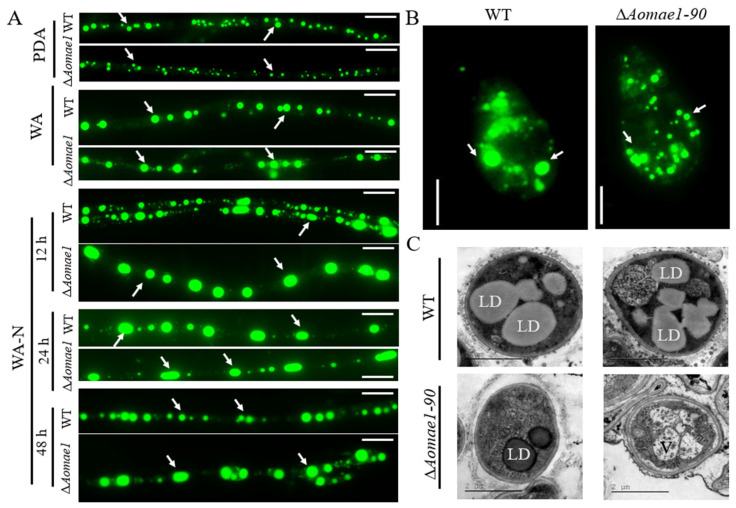
Observation of LD accumulation in the mycelia and conidia of the WT and ∆*Aomae1* mutant strains. The mutant isolate ∆*Aomae1-90* was used as a representative strain. (**A**) BODIPY staining under different culture conditions, scale bar = 5 μm. The WT and mutant strains were stained with BODIPY for observation of the LD morphology after five days of incubation in PDA, WA, and WA-N media. (**B**) LD staining for conidia, scale bar = 5 μm. The arrows in (**A**,**B**) indicate the LDs. (**C**) Observation of LDs via TEM; the WT and mutant strains were incubated on a PDA medium. LD, lipid droplet; V, vacuole.

The morphology of LDs was observed via BODIPY staining after culturing the strain for five days on PDA and WA media. The volume of LDs in the mycelial cells and conidia of ∆*Aomae1* was smaller than that found in the WT strain on the PDA medium, but there was no obvious difference in the number and volume of LDs when they were incubated on the WA medium (Figure 3A,B). The TEM images also showed that the volume of LDs in the WT strain was greater than that in the ∆*Aomae1* mutant incubated on the PDA medium (Figure 3C). In addition, we observed changes in the LDs after the mycelia were induced with nematodes: at 12 h, the LDs of the ∆*Aomae1* mutant were rounded and their volume was greater than that of the WT strain; at 24 h, the volume of LDs of the WT strain was much greater than it was at 12 h, and the LDs of the ∆*Aomae1* mutant became claviform; and at 48 h, the volume of LDs of WT strain decreased to the level observed at 12 h, and the LDs of the ∆*Aomae1* mutant recovered their rounded shape (Figure 3A).

### 3.5. AoMae1 Regulates Sporulation, MDH Activity, and ATP Level

**Figure 4:**
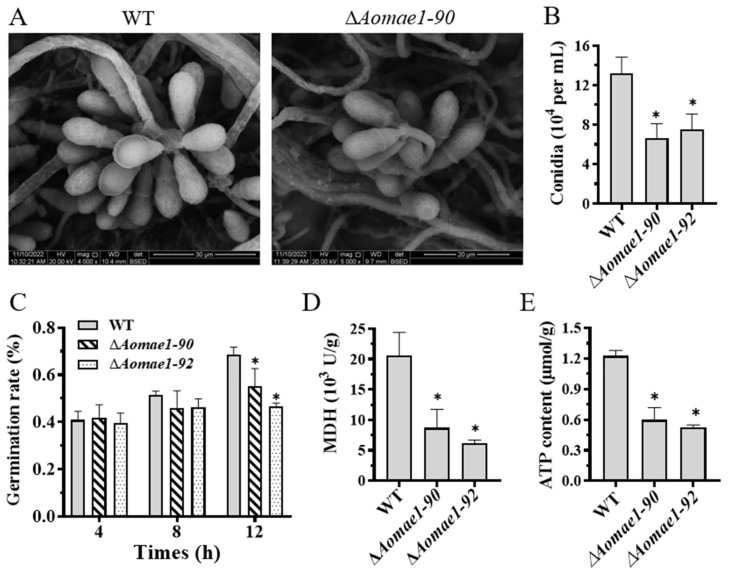
Comparison of conidiation, MDH activity, and ATP content. (**A**) Conidial morphology observation using SEM. (**B**) Comparison of conidia yield. (**C**) Comparison of spore germination rate. (**D**) MDH activity assay. (**E**) ATP content assay. An asterisk (**B**–**E**) indicates a significant difference between the ∆*Aomae1* mutant and the WT strain (Tukey’s HSD, * *p* < 0.05).

Compared with the WT strain, the deletion of *Aomae1* resulted in a reduction in the number of conidiophores (Figure S3), and SEM images showed that the partial conidial morphology of the ∆*Aomae1* strain became longer (Figure 4A). After 14 days of incubation in CMY medium, the total number of spores was counted, and the results showed that the number of conidia of the ∆*Aomae1* strain was significantly reduced (*p* < 0.05) (Figure 4B). In addition, there was no difference in the spore germination rate of the WT and ∆*Aomae1* mutant strains at 4 h and 8 h, whereas the spore germination of the ∆*Aomae1* mutants was significantly reduced at 12 h (*p* < 0.05) (Figure 4C).

In addition, we determined the MDH activity and ATP content. The MDH activity of the Δ*Aomae1* strain was significantly reduced compared with the WT strain (*p* < 0.05) (Figure 4D), and the ATP content of the Δ*Aomae1* strain was considerably lower than that of the WT strain (*p* < 0.05) (Figure 4E).

### 3.6. AoMae1 Regulates Trap Formation, Pathogenicity, and Proteolytic Activity

**Figure 5:**
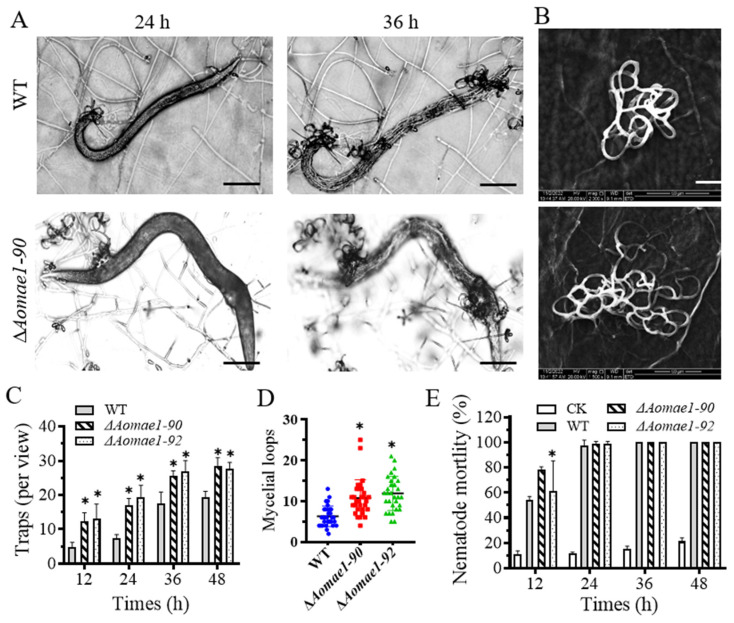
Comparison of trap formation and pathogenicity. (**A**) Representative images of trap formation and nematode predation at 24 and 36 h, scale bar = 100 μm. (**B**) Comparison of the trap morphology of each strain at 48 h, scale bar = 20 μm. (**C**) Comparison of the number of traps. (**D**) Comparison of mycelial loops. A total of 30 random traps were used to determine the mycelial loops. (**E**) Comparison of nematode mortality. An asterisk (**C**–**E**) indicates a significant difference between the ∆*Aomae1* mutant and the WT strain (Tukey’s HSD, * *p* < 0.05). CK indicates the natural death rate of nematodes incubated on the WA medium.

When induced with nematodes for 12 h, the ∆*Aomae1* mutant formed more mature traps, whereas the WT strain only produced a few immature traps. At 24–48 h, there were still considerably more traps in the ∆*Aomae1* mutant than in the WT strain; for example, the WT and ∆*Aomae1* mutant strains produced 17.6 and 26.3 traps per view, respectively, at 36 h, while they produced 19.4 and 28 traps per view, respectively, at 48 h (Figure 5A–D). In addition, the traps produced by the ∆*Aomae1* mutant contained more mycelial loops (4–25) than that of the WT strain (2–13) at 48 h (Figure 5B,C). On average, the ∆*Aomae1* strain produced 9.7 and 8.7 more mature traps per view than the WT strain at 36 h and 48 h, respectively (Figure 5C). In addition, more nematodes were captured by the ∆*Aomae1* mutant at 12 h, whereas all nematodes were captured by the WT and ∆*Aomae1* mutant strains at 24 h after induction (Figure 5E). In addition, the proteolytic activity of the ∆*Aomae1* mutant was lower than that of the WT strain (Figure S2C).

### 3.7. AoMae1 Regulates the Secondary Metabolism

**Figure 6:**
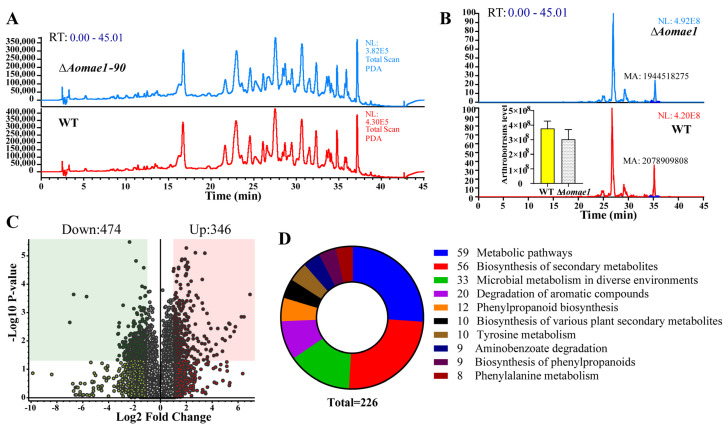
Comparison of the metabolic profiling between WT and ∆*Aomae1* mutant strains. (**A**) Comparison of the high-performance liquid chromatography profiles of the WT and ∆*Aomae1* mutant strains. (**B**) Comparison of the chromatograms of arthrobotrisin anion peaks. The histogram shows that the arthrobotrisin content was comparable to the peak area of the WT and ∆*Aomae1* mutant strains. (**C**) Volcano plot of differentially expressed metabolites between WT and ∆*Aomae1* mutants. (**D**) The enriched KEGG pathways of differentially expressed metabolites.

The extracts of the WT and ∆*Aomae1* mutant strains were analyzed using LC-MS. After a comparison of the chromatograms, we found that the ∆*Aomae1* mutant showed a different characteristic peak at 36 min compared to the WT strain (Figure 6A). In addition, arthrobotrisins, specific metabolites produced by *A. oligospora* and other NT fungi, were analyzed and the peak area of the arthrobotrisins decreased by 19.8% in the ∆*Aomae1* mutant compared with the WT strain (Figure 6B). Metabolic data analysis revealed that 474 compounds were downregulated, and 346 compounds were upregulated in the ∆*Aomae1* mutant versus the WT strain (Figure 6C). KEGG enrichment analysis revealed that 226 differential compounds were highly enriched in the metabolic pathways, including the biosynthesis of secondary metabolites, microbial metabolism in diverse environments, degradation of aromatic compounds, phenylpropanoid biosynthesis, tyrosine metabolism, and biosynthesis of various plant secondary metabolites (Figure 6D).

## 4. Discussion

MDH is an intermediate catalytic enzyme in the TCA cycle, which catalyzes the conversion of malate to oxaloacetate while producing NADH, which plays an essential role in energy metabolism. Like other Mae1 orthologs, AoMae1 contains two conserved domains and shares a moderate degree of similarity with other orthologs from filamentous fungi, suggesting that Mae1 orthologs are highly conserved during evolution. Due to its role in energy metabolism, AoMae1 may have multiple effects on mycelia growth and development. As mentioned above, AoMae1 has pleiotropic functions in the lifecycle of *A. oligospora*, such as hyphal fusion, nuclei, lipid droplet accumulation, sporulation, trap formation, and secondary metabolism.

Cellular communication is essential for the formation of interconnected multi-nuclear filamentous fungi that construct networks by hyphal or conidial fusion [36]. Fusion is a highly dynamic and regulated process, and *ham5* plays an important role as a mitogen-activated protein kinase scaffold during cell fusion in *N. crassa* [37]. In *A. oligospora*, the deletion of *Aoste12* resulted in increased hyphal fusion [38]. In contrast, the knockout of AoMae1, a protein that interacts with AoSte12, resulted in reduced fusion in a nutrient-deprived medium but had no effects on hyphal fusion in a nutrient-rich medium. The inconsistent results of hyphal fusion in different media indicate that AoMae1 has a regulatory effect on hyphal fusion under low nutrient conditions and that this process may be regulated by external substances, such as nutrients. It has been proposed that the trap formation of NT fungi is an evolutionary response of cellulolytic or lignin-degrading fungi to nutrient deficiencies in nitrogen-limiting habitats [10]. Therefore, nutrients are crucial for regulating the hyphal fusion and lifestyle transition of NT fungi. 

Energy metabolism plays a vital role in the mycelial growth and development of fungi. In *Arabidopsis thaliana*, MDH has key functions, regulating embryonic development and heterotrophic metabolism as well as energy balance during seed development [39]. In *A. oligospora*, both ATP content and MDH activity were consistently impaired in the ∆*Aomae1* mutant, suggesting that AoMae1 may be involved in energy metabolism. Although the deletion of *Aomae1* had no obvious influence on mycelial growth, the ∆*Aomae1* mutant incubated on PDA medium had more septa than the WT strain, whereas when the WT and ∆*Aomae1* mutant strains were incubated on WA medium there was no difference in the hyphal septa; additionally, more mycelial septa were observed when the WT and ∆*Aomae1* mutant strains were incubated on WA-N medium, suggesting that nutritional ingredients can impair the ability of AoMae1 to regulate mycelial septa. Meanwhile, the mycelial cell lengths became shorter in the WT and ∆*Aomae1* mutant strains in comparison with those found under nutrient-rich conditions (Figure 2A,B). At the same time, the trap cells were shorter than the hyphal cells, indicating that the cell septum may be related to trap formation. In addition, the absence of *Aomae1* caused a considerable reduction in the number of nuclei. These results suggested that AoMae1 may regulate ATP content by impairing MDH activity, leading to an imbalance in energy metabolism and thus the impairment of various cellular events in *A. oligospora*.

Trap formation is an energy-consuming process that was enhanced under low nutrient and nematode signal induction conditions [40]. LDs are storage organelles at the center of lipid and energy homeostasis, they can associate with most other cellular organelles through membrane contact sites. Importantly, LD biogenesis and degradation, as well as their interactions with other organelles, are tightly coupled to cellular metabolism and are critical to buffer the levels of toxic lipid species [41]. Interestingly, the volumes and sizes of LDs in WT and ∆*Aomae1* mutant strains dynamically altered during trap formation and nematode predation; for example, at the early stage of trap formation (12 h), the mycelial cells of the WT strain consisted of small and large LDs, and at the nematode predation stage (24 h), the volume of LDs was considerably larger than that at 12 h, whereas at the later stage of predation, the LDs returned to volumes similar to those observed without nematode induction. In addition, the volume of LDs in the ∆*Aomae1* mutant considerably exceeded those of the WT strain at the early stage of trap formation, which is consistent with the higher nematode predation efficiency of the ∆*Aomae1* mutant at 12 h. These results suggested that AoMae1 plays a role in the distribution of septa and lipid metabolism, thus regulating the mycelial development, trap formation, and pathogenicity of *A. oligospora*. 

Previous studies have suggested that the glyoxylate cycle has an important role in the pathogenicity of plant pathogenic fungi [42]. The glyoxylate cycle is involved in pleiotropic phenotypes and the antagonism and induction of plant defense responses in *Trichoderma atroviride* [43]. The TCA cycle contributes to CO_2_ sensing and mycelial growth in *Candida albicans*, thereby having a role in its pathogenicity [5]. In *Gibberella zeae*, the deletion of isocitrate lyase Icl caused defects in the formation of sexual fruiting bodies and pathogenicity [44]. In this study, the absence of *Aomae1* resulted in a remarkable increase in traps, and several traps produced by the ∆*Aomae1* mutant had more mycelial loops. Interestingly, the deletion of *Aoste12* resulted in a reduction in trap formation, whereas the traps of the ∆*Aoste12* mutant consisted of more mycelial loops [38]. However, trap formation was impaired in the Δ*Aomls* mutant, which produced only immature traps containing one or two rings [21]. These results suggested that AoSte12 and its interacting protein AoMae1 can regulate trap morphogenesis, such as the number of mycelial loops, whereas their regulatory mechanism needs to be studied further.

In addition to the traps, conidia are also important for the survival and pathogenicity of NT fungi [45]. In *A. oligospora*, the deletion of malate synthase AoMls caused a considerable reduction in conidiation, and the conidia of the Δ*Aomls* mutant were unable to germinate on minimal medium supplemented with sodium oleate [21]. Similarly, the deletion of *Aomae1* resulted in a significant reduction in conidia yield. The above results indicate that the enzymes involved in the glyoxylate and TCA cycles have a vital role in maintaining the sporulation and pathogenicity of pathogenic fungi. In addition, metabolome analysis showed that the absence of *Aomae1* caused the differential expression of 800 compounds and a decrease in the peak area of arthrobotrisins in the ∆*Aomae1* mutant, which is closely related to mycelial development and trap formation [46,47]. Our results suggested that AoMae1 may regulate secondary metabolism by impairing the ATP level but the mechanism needs to be studied further. Therefore, AoMae1 plays a crucial role in conidiation and a role in the secondary metabolism of *A. oligospora.*

## 5. Conclusions

Our results indicate that AoMae1 regulates MDH activity and ATP content, thereby contributing to LD accumulation and energy metabolism, impairing the septa, nuclei, and hyphal fusion, leading to a notable reduction in conidia yield, and regulating trap formation, which, in turn, causes changes in the pathogenicity of *A. oligospora*. Our results revealed the function of AoMae1 in the mycelial development, trap formation, and pathogenicity of *A. oligospora* and provide a good basis for understanding the role of the TCA cycle in the growth and development of NT and other filamentous fungi.

## Data Availability

Not applicable.

## Supplementary Materials

The following supporting information can be downloaded at: https://www.mdpi.com/article/10.3390/jof9040496/s1. Figure S1: Phylogenetic analysis and validation of Aomae1 knockout strain; Figure S2: Trap morphology and extracellular protease activity; Figure S3: Observation of conidiophores and sporulation of the WT and mutants on CMY medium; Table S1: Information of the plasmids used in this study; Table S2: List of primers used in this study.
