# Supplementary material for: AoMae1 Regulates Hyphal Fusion, Lipid Droplet Accumulation, Conidiation, and Trap Formation in Arthrobotrys oligospora
Source: J Fungi (Basel). 2023 Apr 21;9(4):496. doi: 10.3390/jof9040496 (PMC10146936; doi:10.3390/jof9040496)
Supplement: Supplementary file 1 [file jof-09-00496-s001.zip › jof-2344712-supplementary.pdf]

Supporting Information

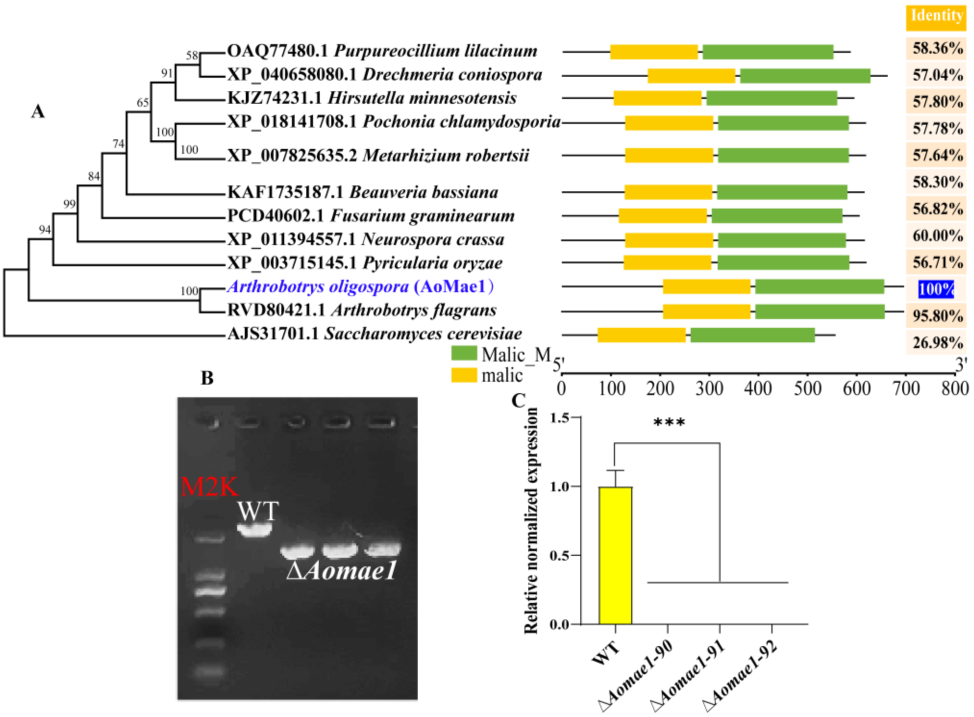

Figure S1. Phylogenetic analysis and validation of *Aomae1* knockout strain. A Phylogenetic and structure domain analysis of Mae1 orthologs from different fungi. (B and C) Validation of knockout strains by PCR (B) and RT-PCR (C). M2K, DNA marker. An asterisk indicates a significant difference between the Δ*Aomae1* mutant and the WT strain (Tukey's HSD, *$p < 0.01$).

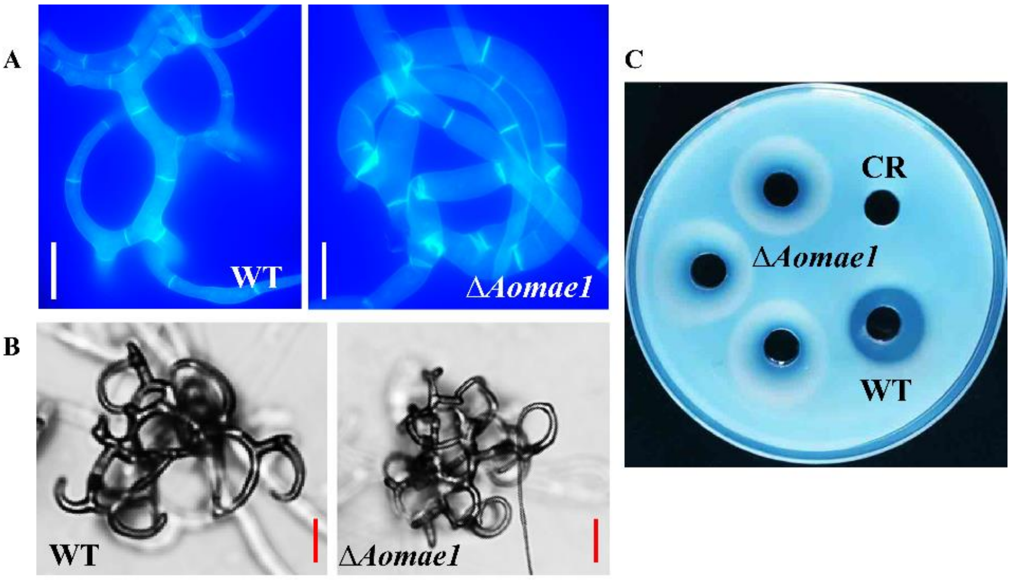

Figure S2. Trap morphology and extracellular protease activity. A. Trap morphology was observed by CFW staining. Scale bar = 10 μm. B. Trap morphology was observed by a light microscopy. Scale bar = 20 μm. C. Comparison of extracellular protease activity. CR, the PD broth was used as a control sample.

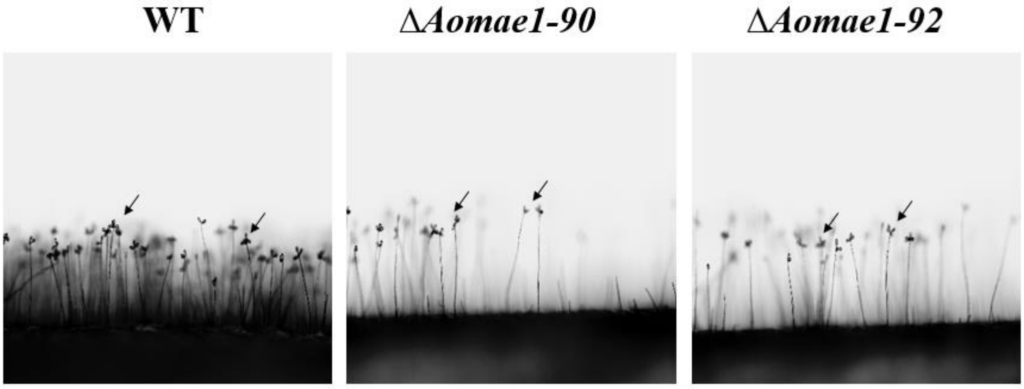

Figure S3. Observation of conidiophores and sporulation the WT and mutants on CMY medium. Black arrows: conidia.

Table S1. Information of the plasmids used in this study.

Plasmids	Selection marker	Application
pCSN44	hph+	For hygromycin resistance
pRS426	Amp+	For constructing the disruption fragment

Table S2. List of primers used for gene manipulation in this study.

Primers	Sequences	Application
AoMae1-5F	GTAACGCCAGGGTTTTCCCAGTCACGACGGTGTGATTTCAGGCGGGGA	Amplify the *Aomae1*
AoMae1-5R	ATCCACTTAACGTTACTGAAATCTCCAACCTCCCATACCTTTGCATTGTCC	gene 5’ flank
AoMae1-3F	CTCCTTCAATATCATCTTCTGTCTCCGACGGGGTGCTTGGTTCTTGGAT	Amplify the *Aomae1*
AoMae1-3R	GCGGATAACAATTTCACACAGGAAACAGCCTATTCCCTGTTTCCCGCGT	gene 3’ flank
Hph-f	GTCGGAGACAGAAGATGATATTGAAGGAGC	Amplify the *hph*
Hph-r	GTTGGAGATTTCAGTAACGTTAAGTGGAT	cassette
Mae1-PF	GGACAATGCAAAGGTATGGGAG	Primers for PCR
Mae1-PR	ATCCAAGAACCAAGCACCCC	verification
Mae1-RF	ATGATTATCCAGTTTGAGGA	Primers for RT-qPCR
Mae1-RR	TGGCAACACCAACACCAGCA	verification
